# Editorial: Enhancing livestock breeding through advanced genetic tools and phenotyping systems

**DOI:** 10.3389/fgene.2025.1617113

**Published:** 2025-05-14

**Authors:** Yulin Bai

**Affiliations:** Key Laboratory of Freshwater Fisheries and Germplasm Resources Utilization, Ministry of Agriculture and Rural Affairs, Freshwater Fisheries Research Center, Chinese Academy of Fishery Sciences, Wuxi, China

**Keywords:** livestock breeding, high-throughput phenotyping, genomic selection, genetic diversity, sustainable agriculture

Livestock breeding stands is undergoing a major transformation, where the integration of advanced genetic tools and precision phenotyping systems offers promising approaches to address global challenges in food security, sustainability, and animal welfare. The Research Topic *Enhancing Livestock Breeding through Advanced Genetic Tools and Phenotyping Systems* explores how genomic technologies, high-throughput phenotyping, and data-driven approaches can accelerate genetic gains in livestock. This Research Topic of six articles presents novel methodologies and their applications across diverse species, providing valuable insights for researchers and breeders aiming to enhance productivity, resilience, and sustainability in animal production systems.

**FIGURE 1:**
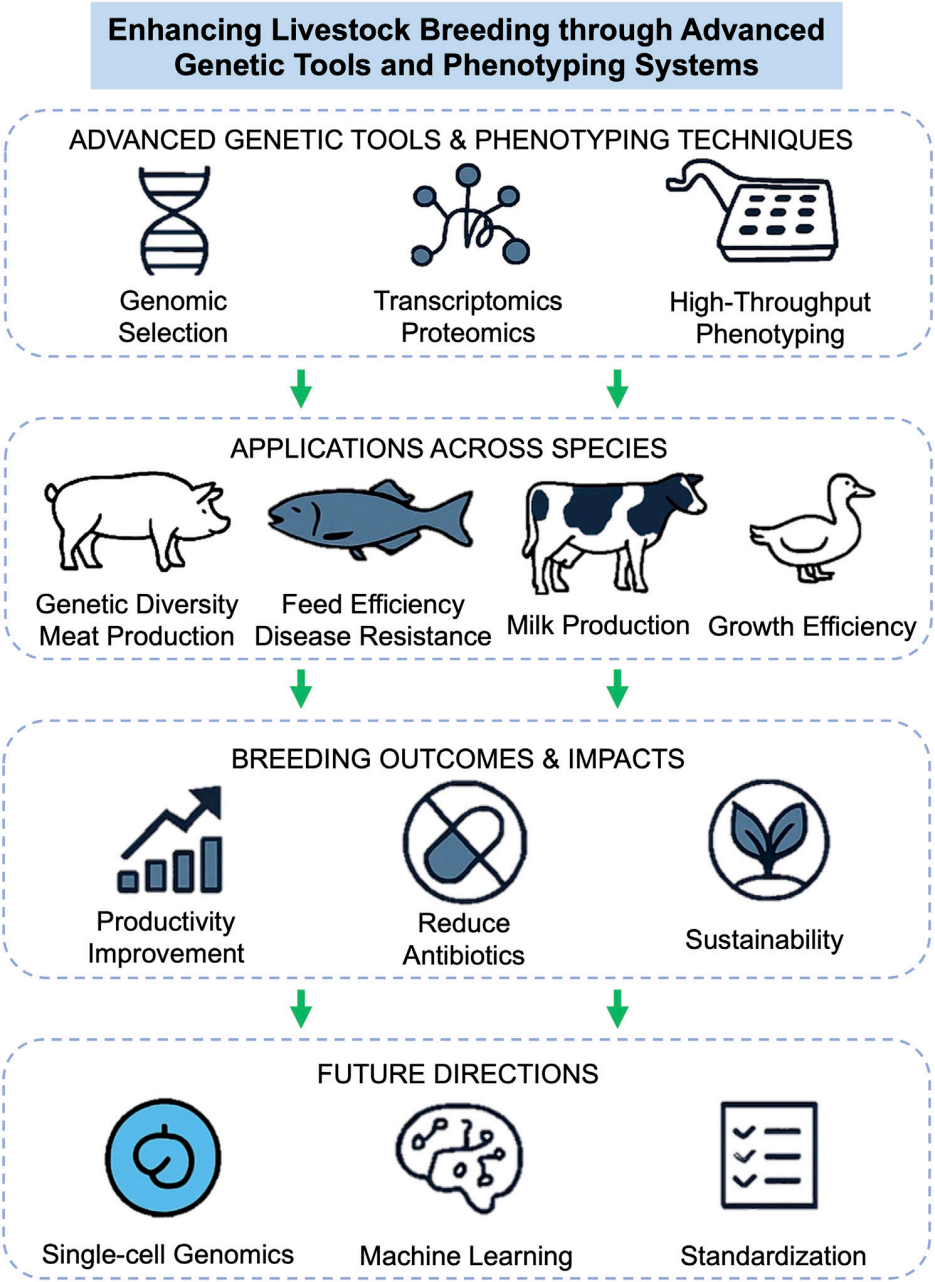
Conceptual framework for enhancing livestock breeding through advanced genetic tools and phenotyping systems.

The primary objective of this Research Topic is to highlight the synergy between advanced genetic tools and phenotyping systems in improving livestock breeding outcomes. By leveraging genomic selection, whole-genome sequencing, and high-resolution phenotyping, researchers can better understand complex traits, optimize breeding programs, and address environmental and economic challenges. The articles in this Research Topic tackle these goals through diverse approaches, ranging from population genetics and transcriptomics to proteomic analyses and disease resistance studies (Figure 1).

The article “*Population Structure and Genetic Diversity of Mi Pigs Based on SINE-RIPs*” investigates the genetic diversity of Mi pigs, a Chinese indigenous breed, using Short Interspersed Nuclear Element-Retrotransposon Insertion Polymorphisms (SINE-RIPs) (Wang et al.). The authors analyze the population structure to identify genetic variations that can inform conservation strategies and breeding programs, emphasizing the importance of preserving genetic diversity in indigenous breeds for sustainable livestock production.

In “*Different Responses of the Intestine and Liver Transcriptome to High Levels of Plant Protein in Diets for Large Yellow Croaker (Larimichthys crocea)*”, the authors explore the transcriptomic responses of large yellow croaker to diets high in plant protein (Ke et al.). By examining gene expression changes in the intestine and liver, this study provides insights into the molecular mechanisms underlying dietary adaptation, offering a foundation for optimizing feed formulations in aquaculture species, which indirectly supports sustainable livestock systems through improved feed efficiency.

The article “*Application of Selection Index for Enhancing Resistance to Cryptocarya irritans and Vibrio alginolyticus in Large Yellow Croaker*” focuses on improving disease resistance in large yellow croaker through genomic selection (Wang et al.). The authors develop a selection index to enhance resistance against *Cryptocarya irritans* and *Vibrio alginolyticus*, two significant pathogens in aquaculture. This work demonstrates the potential of genetic tools to improve health outcomes, reducing reliance on antibiotics and supporting sustainable fish farming practices.

The study “*Whole-Genome Re-Sequencing Study on Body Size Traits at 10-Weeks of Age in Chinese Indigenous Geese*” employs whole-genome re-sequencing to investigate the genetic basis of body size traits in Chinese indigenous geese at 10 weeks of age (Sun et al.). By identifying key genomic regions associated with growth, the authors provide a foundation for marker-assisted selection, enabling breeders to enhance growth efficiency in geese, a species of growing importance in poultry production.

In “*Genome-Wide Association Studies for Milk Production Traits and Persistency of First Calving Holstein Cattle in Türkiye*”, the authors conduct a genome-wide association study (GWAS) to identify genetic markers linked to milk production traits and persistency in Holstein cattle (Erdoğan et al.). This study highlights the application of genomic tools in dairy cattle breeding, offering a pathway to improve milk yield and lactation persistency, which are critical for economic sustainability in dairy farming.

Finally, “*Differential Analysis of Ubiquitin-Proteomics in Skeletal Muscle of Duroc Pigs and Tibetan Fragrant Pigs*” examines the ubiquitin-proteome in the skeletal muscle of Duroc and Tibetan fragrant pigs (Li et al.). By analyzing differences in protein ubiquitination, the authors uncover molecular mechanisms underlying muscle development and meat quality, providing insights that can guide breeding strategies to improve carcass traits and meat production in pigs.

Overall, these articles reveal the significant potential of integrating advanced genetic tools with phenotyping systems in livestock breeding. They address a wide range of species—pigs, geese, cattle, and fish—highlighting the robustness of these technologies across different production systems. The studies also underscore the importance of tailoring breeding strategies to specific traits, such as disease resistance, growth, and feed efficiency, while considering the unique genetic makeup of each species or breed.

The findings in this Research Topic have broader implications for the livestock industry. For instance, improving disease resistance in aquaculture species, as shown in the large yellow croaker studies, can reduce economic losses and promote sustainable practices. Similarly, enhancing milk production traits in Holstein cattle and preserving genetic diversity in indigenous breeds like Mi pigs contribute to global food security and biodiversity conservation. However, challenges remain, including the high cost of genomic and phenotyping technologies and the need for standardized protocols to ensure data reproducibility across studies.

Looking ahead, the integration of emerging technologies, such as single-cell genomics, breeding microarrays, and machine learning could further enhance the precision of livestock breeding programs. Collaborative efforts between researchers, breeders, and policymakers will be crucial to make these technologies accessible, particularly in developing regions where livestock production is a cornerstone of livelihoods. This Research Topic provides a foundation for future research and innovation, encouraging the adoption of advanced tools to address specific challenges in global agriculture, such as enhancing climate resilience in breeding programs, improving feed efficiency, and standardizing data collection across phenotyping platforms.

We express our gratitude to the authors for their valuable contributions and to the reviewers for their rigorous evaluations. We hope this Research Topic inspires continued research and practical applications that advance the future of livestock breeding.

